# Ten Years of Addressing Children’s Health through Regulatory Policy at the U.S. Environmental Protection Agency

**DOI:** 10.1289/ehp.11390

**Published:** 2008-09-30

**Authors:** Devon Payne-Sturges, Debra Kemp

**Affiliations:** 1 Office of Children’s Health Protection and Environmental Education, U.S. Environmental Protection Agency, Washington, D.C., USA; 2 Environment and Resources Division, Abt Associates Inc., Bethesda, Maryland, USA

**Keywords:** children’s environmental health, EPA’s policy on evaluating health risks to children, Executive Order 13045

## Abstract

**Background:**

Executive Order (EO) 13045, Protection of Children From Environmental Health Risks and Safety Risks, directs each federal agency to ensure that its policies, programs, activities, and standards address disproportionate environmental health and safety risks to children.

**Objectives:**

We reviewed regulatory actions published by U.S. Environmental Protection Agency (EPA) in the *Federal Register* from April 1998 through December 2006 to evaluate applicability of EO 13045 to U.S. EPA actions and consideration of children’s health issues in U.S. EPA rulemakings.

**Discussion:**

Although virtually all actions discussed EO 13045, fewer than two regulations per year, on average, were subject to the EO requirement to evaluate children’s environmental health risks. Nonetheless, U.S. EPA considered children’s environmental health in all actions addressing health or safety risks that may disproportionately affect children.

**Conclusion:**

The EO does not apply to a broad enough set of regulatory actions to ensure protection of children’s health and safety risks, largely because of the small number of rules that are economically significant. However, given the large number of regulations that consider children’s health issues despite not being subject to the EO, other statutory requirements and agency policies reach a larger set of regulations to ensure protection of children’s environmental health.

In 1997 President Clinton signed Executive Order (EO) 13045, Protection of Children from Environmental Health Risks and Safety Risks (Clinton 1997). This order directs each federal agency to “make it a high priority to identify and assess environmental health risks and safety risks that may disproportionately affect children” and to “ensure that its policies, programs, activities, and standards address disproportionate risks to children that result from environmental health risks or safety risks” (Clinton 1997). That same year, the U.S. Environmental Protection Agency (EPA) created the Office of Children’s Health Protection to support its implementation of the Executive Order and its National Agenda to Protect Children’s Health from Environmental Threats (U.S. EPA 1996). Finally, U.S. EPA created the Children’s Health Protection Advisory Committee (CHPAC) in 1997, which is an independent body that advises U.S. EPA on regulations, research, and communication issues relevant to children. These actions were taken in direct response to the growing body of scientific knowledge that demonstrated that children may suffer disproportionately from environmental health and safety risks [National Research Council (NRC) 1993, 1994]. Disproportionate risks to or impacts on children may occur when children are more sensitive to, are more likely to be exposed to, or are likely to be exposed to higher levels of a particular pollutant or agent being considered in the rulemaking than adults are (U.S. EPA 2006c).

Although EO 13045 has been pointed to as a model policy because it specifically addresses children and directs federal agencies to deliberately consider disproportionate risks to children (Fischer 2007), others say that the impact of EO has waned over the years (Carlson 2005; Spady 2006). The U.S. EPA recently marked the tenth anniversary of EO 13045, and CHPAC concluded that children’s environmental health considerations are still not explicitly addressed in U.S. EPA decisions, policies, and programs and urged renewed commitment to EO 13045 (Johnson 2007; Marty 2007). In this article we review U.S. EPA regulatory actions to evaluate applicability of EO 13045 and consideration of children’s health issues in U.S. EPA rulemakings.

## Requirements to consider children’s health in regulatory development at U.S. EPA

In addition to EO 13045, U.S. EPA has several policies that seek to ensure that children’s health is adequately analyzed and addressed in regulatory actions. These policies are often complemented by specific statutory requirements that direct U.S. EPA to further evaluate and protect children’s environmental health.

## Executive Order 13045

Signed in 1997, EO 13045 establishes a federal-wide policy for identifying, assessing, and addressing disproportionate health and safety risks to children (Clinton 1997). Section 5 establishes requirements for regulations that *a*) are initiated after 21 April 1997 or proposed after 21 April 1998, *b*) are economically significant according to EO 12866 (Clinton 1993), and *c*) concern an environmental health or safety risk that an agency has reason to believe may disproportionately affect children. For these actions, the agency must submit to the Office of Management and Budget (OMB) an evaluation of the environmental health or safety effects of the planned regulation on children and an explanation of why the planned regulation is preferable to other alternatives considered. The policy does not, however, direct a federal agency to select a particular outcome.

## U.S. EPA children’s environmental health policies

Two years before President Clinton signed EO 13045, U.S. EPA announced its Policy on Evaluating Health Risks to Children (U.S. EPA 1995), which requires that U.S. EPA consistently and explicitly evaluate health risks to infants and children in all risk assessments, risk characterizations, and environmental and public health standards. The policy states that to the “degree permitted by available data in each case, the Agency will develop a separate assessment of risks to infants and children or state clearly why this is not done” (U.S. EPA 1995). This policy applies whether or not risks to children are disproportionate or different from risks to adults (U.S. EPA 2006c).

Consideration of children’s environmental health risks is also incorporated in U.S. EPA guidance documents that explain the action development process and establish consistent practices (U.S. EPA 2003a, 2004, 2005d, 2005f, 2006a). In 1998, U.S. EPA published a rule-writer’s guide to EO 13045 (U.S. EPA 1998), which was revised in 2006 (U.S. EPA 2006c). The *Guide to Considering Children’s Health When Developing EPA Actions: Implementing Executive Order 13045 and EPA’s Policy on Evaluating Health Risks to Children* (U.S. EPA 2006c) reflects new developments in risk assessments, regulatory policy, and action development, and more clearly integrates the U.S. EPA Children’s Health Policy with the overall action development process.

### Statutory requirements to consider children’s environmental health

Several statutes under which the U.S. EPA promulgates regulations require consideration of children or other susceptible populations. Examples include the Clean Air Act (1970), Federal Food, Drug, and Cosmetic Act (FFDCA; 1938), and the Safe Drinking Water Act (1996).

The Clean Air Act of 1970, as amended, requires U.S. EPA to set National Ambient Air Quality Standards (NAAQS) at levels that protect public health, including susceptible populations, with an “adequate margin of safety” [Clean Air Act 1970, 42 U.S.C. §7409 (b)(1)]. Other air quality requirements, such as those to control emissions of hazardous air pollutants, are based on technological performance, not human health risk. Risks to public health and impacts on the environment are not typically considered in the development of national emissions standards for hazardous air pollutants (NESHAPs); rather, the residual risk program considers risks and impacts within 8 years, after U.S. EPA issues an air toxics standard for a given source category (U.S. EPA 2003e). However, if allowed by statute, U.S. EPA may consider whether a particular pollutant affects human health and disproportionately affects children when determining whether a technology-based standard should be set at a level that is more stringent than a level based solely on the performance of technology (U.S. EPA 2006c).

The FFDCA of 1938, as amended by the Food Quality Protection Act of 1996 (FQPA), requires U.S. EPA to consider exposure of infants and children when establishing a tolerance (a limit on the amount of pesticides that may remain in or on foods). Specifically, the statute requires that U.S. EPA “ensure that there is a reasonable certainty that no harm will result to infants and children from aggregate exposure to the pesticide chemical residue” [FQPA 1996, 21 U.S.C. §346(b)(2)(A)(i)]. In addition, U.S. EPA must apply an additional 10-fold margin of safety for infants and children to the tolerance limit unless it determines that a different margin of safety, based on reliable data, will be safe for infants and children.

The Safe Drinking Water Act of 1974, as amended in 1996, requires U.S. EPA to set regulations to avoid any known or anticipated adverse human health effects and allow an adequate margin of safety. The 1996 amendments required U.S. EPA to consider the effects of contaminants upon groups of individuals who may be more sensitive than the general population when setting standards (Safe Water Drinking Act Amendments 1996, 42 U.S.C. §300g-1).

## Methods

In preambles, U.S. EPA documents the basis of the rule and addresses all executive orders and statutory requirements. We reviewed preambles to proposed rules, final rules, and direct final rules that U.S. EPA published by in the *Federal Register* from April 1998 (effective date of EO 13045) through December 2006 (end of the study period). We excluded administrative or site-specific actions, including minor regulatory corrections or amendments, procedural regulations, extensions of dates for public comment or compliance, updates to the National Priorities List, approvals of state air quality implementation plans, and designations of air quality planning areas. If a regulation was proposed and finalized within the study period, only the final action was considered.

Consistent with the EO 13045 applicability criteria, we determined if each action *a*) was economically significant; *b*) was health-or safety-based; *c*) addressed a health or safety risk that U.S. EPA has reason to believe may disproportionately affect children; *d*) discussed children’s health impacts; and *e*) discussed applicability of EO 13045.

To determine economic significance, we relied on the definition provided in EO 13045, which refers to EO 12866–Regulatory Planning and Review (Clinton 1993). *Federal Register* notices generally include a statement on economic significance. For notices that did not provide an explicit discussion of economic significance, we referred to the Unified Agenda of the Federal Regulatory and Deregulatory Actions (Regulatory Information Service Center 2008) and/or contacted the rule writer directly for clarification.

U.S. EPA interprets EO 13045 as applying only to regulatory actions concerning health or safety risks where the analysis it requires has the potential to influence the regulation (U.S. EPA 2006c). Neither EO 13045 nor U.S. EPA defines “health- or safety-based” rules, but U.S. EPA does discuss applicability of EO 13045 to various types of actions (U.S. EPA 2006c). Consistent with U.S. EPA guidance, health- or safety-based rules do not include actions based solely on technology performance; sampling methodologies and test procedures; procedural rules; ecologic standards unrelated to human health; information-gathering rules; permit application rules; individual state program approval decisions; state implementation plan and federal implementation plan rules; or rules implementing specific standards established by Congress. Where it was not explicitly stated in the U.S. EPA action, we used the above guidelines to determine if each was a health- or safety-based action.

By performing keyword searches of relevant terms (e.g., child, infant, toddler, adolescent), we reviewed each *Federal Register* notice to determine whether U.S. EPA believed that an action addressed an environmental health or safety risk that may have a disproportionate impact on children.

Any action for which the *Federal Register* notice discussed (even briefly) the effects of the regulation or the regulated pollutant on children’s health was classified as considering the impacts on children’s environmental health. For example, an action might discuss the risks to children from exposure to the regulated pollutant, model health risks to children from exposure to a regulated pollutant, or estimate the number of households where children’s exposure to a regulated pollutant would be brought below a level of concern. This approach was chosen instead of evaluating in detail compliance with the administrative requirements of EO 13045 for specific actions (e.g., such as submission of information to OMB or a discussion of alternatives) or with other policies because of the large number of regulations in the study period and the availability of specific information in rulemaking dockets.

## Results

**Figure 1:**
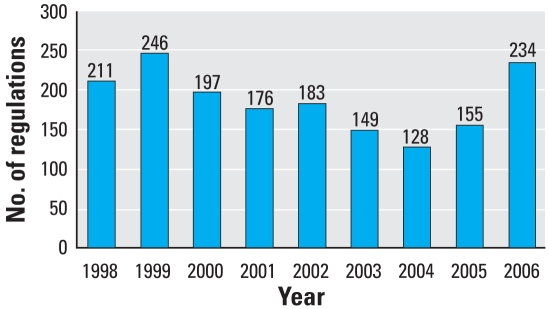
Number of regulations evaluated, 1998 through 2006.

**Figure 2:**
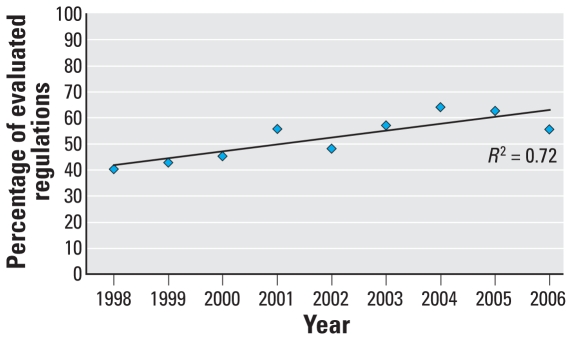
Percent of evaluated regulations that considered children, April 1998 through December 2006.

We evaluated 1,679 regulatory actions, including 132 proposed rules, 1,438 final rules, and 109 direct final rules. Figure 1 summarizes the number of actions in each year included in this analysis. Of these regulations, 860 (51%) explicitly considered children’s health. Figure 2 illustrates the percentage of regulations that considered children’s health in each year from April 1998 through December 2006, which shows a modest increase in the consideration of children over time.

**Figure 3:**
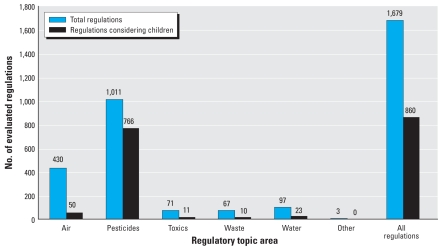
Evaluated regulations that considered children’s health compared with the total number of regulations, by topic area.

Figure 3 shows the total number of regulations and the regulations that considered impacts on children, by environmental topic. Children’s health was considered most frequently in pesticide regulations (766 of 1,011; 74%), almost all of which (995; 98%) related to tolerances. Eighty-five of the tolerance actions were health- or safety-based regulations, and 76% of the tolerance actions explicitly discussed children’s health. Fifty (12%) of the 430 air regulations explicitly considered children’s health, of which 29 (58%) were health- or safety-based regulations and 15 (30%) concerned an environmental health risk that poses a disproportionate risk to children. One hundred seventeen (27%) of the 430 air regulations were considered health- or safety-based actions. Of the thirteen NAAQS regulations, six included explicit consideration of children’s health. The other NAAQS regulations stated that the actions *a*) did not establish an environmental standard intended to mitigate health or safety risks (U.S. EPA 2000b, 2002b); *b*) implemented a previously promulgated health-based federal standard (U.S. EPA 2005c); *c*) removed a no longer applicable portion of a health- or safety-based standard (U.S. EPA 2000a); or *d*) did not involve the consideration of relative environmental health or safety risks (U.S. EPA 2005a). Children’s health considerations were included in regulations pertaining to other media as follows: 15% of toxics, 15% of waste, and 24% of water regulations.

**Figure 4:**
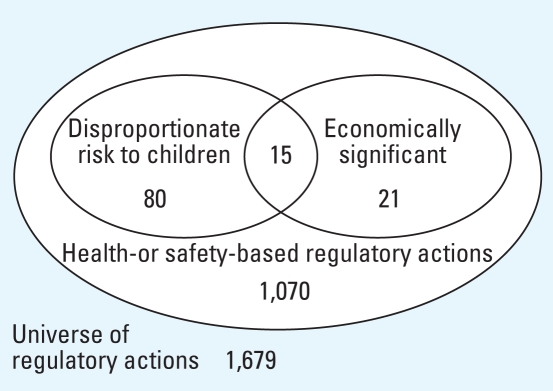
Characteristics of evaluated regulations.

Figure 4 illustrates the number of health- or safety-based actions, including those that also concerned a health or safety risk that might disproportionately affect children and those that were economically significant. Of the actions evaluated, 1,070 (64%) were considered health-or safety-based actions. Of these, 80 (7%) addressed health or safety risks that may disproportionately impact children, all of which considered children’s health as part of the regulatory decision making process. Fifty (63%) of these 80 actions related to establishing, revoking, or amending pesticide tolerances.

Twenty-four of the 43 economically significant actions included an evaluation of children, of which 19 were health- or safety-based actions. Fifteen actions were health- or safety-based, concerned a health or safety risk that might disproportionately affect children, and were economically significant. Of these, 12 were subject to EO 13045, as discussed below.

Of the 860 regulations that explicitly considered children’s health, < 2% were subject to EO 13045 (Table 1). These 13 actions represent 15% of the 89 actions that addressed health or safety risks that may disproportionately impact children. The Air Quality Index Reporting rule was subject to EO 13045 despite not being economically significant (U.S. EPA 1999a). OMB advised U.S. EPA that the regulation should otherwise be considered a “significant regulatory action,” per EO 12866 (Clinton 1993), which makes for a total of 14 actions subject to EO 13045.

The Emission Standards from Heavy-Duty Highway Engines and Vehicles was primarily a technology-based regulation, but was subject to EO 13045 (U.S. EPA 2000a). The U.S. EPA considered the toxicity of a pollutant and its impacts on children’s health, while also considering costs and other relevant factors (U.S. EPA 2000a). Further, the U.S. EPA noted that children’s health was addressed in the NAAQS rulemakings for ozone and particulate matter, which establish the maximum permissible concentrations of the most common air pollutants that other air regulations help meet (U.S. EPA 2000a).

Three National Primary Drinking Water regulations would have been subject to EO 13045, except they were initiated or proposed before the effective dates in EO 13045. Other regulations—such as the Effluent Limitations Guidelines and New Source Performance Standards for the Metal Products and Machinery Point Source Category and the National Primary Drinking Water Regulations: Ground Water Rule—were subject to EO 13045 when they were proposed but not when they were finalized because they were no longer economically significant (U.S. EPA 2003b, 2006g).

In addition to not meeting the applicability criteria, U.S. EPA also determined that actions were not subject to EO 13045 for some of the following reasons: *a*) No alternative technologies existed that would provide greater stringency at a reasonable cost, so any such analysis would not affect the decision (U.S. EPA 2003d); *b*) U.S. EPA did not have the data necessary to conduct such analysis and cannot obtain such data with available resources (U.S. EPA 1999b); *c*) the rule implemented a Congressional directive (U.S. EPA 1999d); and *d*) the effects on children’s health were considered in previous regulations setting NAAQS levels (U.S. EPA 2002b).

## Discussion

EO 13045 is thought to have a significant impact on U.S. EPA regulations and policy initiatives (Bergeson 2005), and others claim that only U.S. EPA adequately evaluates impacts on children as required by EO 13045 (Spady 2006). Despite this praise for EO 13045, our analysis suggests that other policies and statutory requirements might be more influential in ensuring that children’s health is evaluated in the regulatory development process (e.g., FQPA). Although 99 percent of the regulations (1,658) reviewed discussed EO 13045, few regulations are actually subject to its requirement to evaluate children’s health risks (less than two regulations per year on average).

Given the EO 13045 applicability criteria, many relevant regulations are not subject to its requirement to evaluate children’s health risks. Only a small number of actions are classified as “economically significant” under EO 12866. Because of this criterion, EO 13045 applied to only 15 of the 80 regulations that were health-or safety-based and addressed a risk that may disproportionately impact children.

Further, because of the somewhat subjective nature of the other EO criteria (i.e., actions that concern a health or safety risk that may disproportionately affect children) and their application, the interpretation of the EO applicability criteria is important. Despite guidance to and efforts toward increasing consistency across U.S. EPA program offices, some regulations during the study period included misinterpretations of the applicability criteria. For example, in its Standards for the Use or Disposal of Sewage Sludge, U.S. EPA commented on the environmental risk posed by the rule itself instead of overall environmental issue or pollutant or environmental contaminant being addressed by the action; U.S. EPA nonetheless evaluated risks to children according to the EPA Children’s Health Policy (U.S. EPA 1999e). Similarly, U.S. EPA misinterpreted the applicability criteria in some regulations by stating that the EO does not apply because the rule “does not establish an environmental standard that is intended to have a negatively disproportionate effect on children” (U.S. EPA 2006h, 2006i). Instead U.S. EPA should have considered the possibility of a negative, disproportionate effect on children from the overall environmental issue or contaminant being addressed by the action, as opposed to the action itself.

Despite its limited application, EO 13045 raises the profile of children’s health in rulemaking and increases the transparency of the regulatory development process. For proposed rules, it provides the opportunity to request peer-reviewed data and analyses to assist U.S. EPA in the evaluation of children’s health impacts. The requirement to discuss EO 13045 in *Federal Register* notices provides a forum for U.S. EPA to discuss other matters related to children’s health, such as the notice for National Emissions Standards for Coke Batteries that included a discussion of the first application of *Supplemental Guidance for Assessing Susceptibility from Early-Life Exposure to Carcinogens* (U.S. EPA 2005e). Similarly, the notice for Mercury Emissions from Mercury Cell Chlor-Alkali Plants cited a recommendation by CHPAC to consider the regulation’s impacts on children (U.S. EPA 2003e).

Given the large number of regulations that consider children’s health issues, despite their not being subject to the Executive Order, other policy drivers clearly reach a broader set of regulations than EO 13045. Some regulations cite the EPA Children’s Health Policy or the National Agenda as the rationale for considering children’s health issues. Unlike EO 13045, which has the scrutiny provided by OMB review and the visibility of a discussion in the *Federal Register,* the EPA Children’s Health Policy cannot be readily evaluated with a review of a *Federal Register* notice because there is no requirement to discuss the policy in the preamble. However, the Children’s Health Policy likely influences a broader set of regulations given that it does not have as limiting applicability criteria as EO 13045.

Our analysis indicates that statutory requirements are major drivers of children’s health evaluation in instances where EO 13045 does not apply. U.S. EPA regulations published under the authority of statutes requiring explicit consideration of children are more likely than other regulations to evaluate children’s health issues. Indeed, U.S. EPA may have considered children in these actions even in absence of other policies because of its statutory obligations. EO 13045 and the EPA Children’s Health Policy provide a framework most relevant to regulations under the authority of statutes that do not require explicit consideration of susceptible populations.

## Conclusion

Executive Order 13045 does not apply to a broad enough set of regulatory actions to ensure maximum protection of children’s health and safety risks, largely because of the small number of rules that are economically significant. Taken together, statutes, executive orders, policies, and guidance provide a framework that encourages and/or requires consideration of risks to children in U.S. EPA regulatory actions. This multipronged approach to addressing children’s health in environmental regulation emphasizes the need for coordination across U.S. EPA to ensure consistent interpretation and application of the respective policies. This combination of children’s health policies also requires a clear commitment to children’s environmental health by all program offices for understanding, following, and implementing the relevant policies or statutes, because each is unique in its applicability, scope, and legal authority.

## Limitations of the Analysis

With this analysis we intended to characterize the consideration of children in U.S. EPA regulations published over a period of > 8 years. Thus, we did not examine the quality or scope of the evaluations or how or if the evaluation influenced regulatory outcome, which would need to be a more focused effort on specific regulations. Further, for regulations that did not include explicit consideration of children, this analysis does not determine whether children’s health should have been evaluated or was evaluated in the supporting regulatory analysis, but not mentioned in the preamble.

## Directions for Future Research

In 2001, the *EPA Task Force Report on Improving EPA Regulations* recommended that U.S. EPA evaluate the effectiveness of the regulatory process in protecting children’s health since EO 13045 was published (U.S. EPA 2001a). This article represents a first step toward evaluating U.S. EPA’s implementation of EO 13045 and related policies. Future research could include evaluating how children’s health considerations influence regulatory outcomes or identifying instances where children’s health is not considered in regulatory actions and should have been. With a better understanding of the historical consideration of children’s health in regulation, one could consider areas where additional policy and guidance may be needed to better protect children’s health, identify the barriers to evaluating the impacts on children, and conduct robust evaluations of the effect of U.S. EPA policies and regulations on children’s health.

## Figures and Tables

**Table 1:** Rules subject to EO 13045.

Regulation	Regulation type	Publication date	Reference
Air quality index reporting	Final	August 1999	U.S. EPA 1999a
Persistent bioaccumulative toxic chemicals; toxic chemical reporting	Final	October 1999	U.S. EPA 1999c
Tier 2 motor vehicle emissions standards and gasoline sulfur control requirements	Final	February 2000	U.S. EPA 2000c
Control of emissions from heavy-duty highway engines and vehicles	Final	October 2000	U.S. EPA 2000a
Lead: identification of dangerous levels	Final	January 2001	U.S. EPA 2001c
Lead: toxic chemical release reporting	Final	January 2001	U.S. EPA 2001d
Heavy-duty engine/vehicle standards and highway diesel fuel requirements	Final	January 2001	U.S. EPA 2001b
Guidelines and standards for concentrated animal feeding operations	Final	February 2003	U.S. EPA 2003c
Electric utility steam-generating units	Final	May 2005	U.S. EPA 2005b
National primary drinking water regulations: long-term 2 enhanced surface water treatment rule	Final	January 2006	U.S. EPA 2006f
Lead: renovation, repair, and painting program	Proposed	January 2006	U.S. EPA 2006d
Control of hazardous air pollutants from mobile sources	Proposed	March 2006	U.S. EPA 2006b
National ambient air quality standards for particulate matter	Final	October 2006	U.S. EPA 2006e
